# Editorial: Neural Mechanisms Underlying Internet Gaming Disorder

**DOI:** 10.3389/fpsyt.2018.00404

**Published:** 2018-09-06

**Authors:** Jin-Tao Zhang, Matthias Brand

**Affiliations:** ^1^State Key Laboratory of Cognitive Neuroscience and Learning and IDG/McGovern Institute for Brain Research, Beijing Normal University, Beijing, China; ^2^Department of General Psychology: Cognition and Center for Behavioral Addiction Research (CeBAR), University of Duisburg-Essen, Duisburg, Germany

**Keywords:** internet gaming disorder, neural mechanism, fMRI, reward processing, executive control function

Internet Gaming Disorder (IGD), a worldwide mental health issue, has been extensively studied over the last two decades and many studies show that IGD shares characteristics with substance use disorders (SUD) and pathologic gambling in etiology, phenomenology, neurobiological mechanisms, and treatment efficacy. Using Magnetic Resonance Imaging (MRI) techniques along with electroencephalographic and event-related potentials (ERPs) methods, a growing number of studies have emerged exploring neural biomarkers of IGD. Based on neuroscientific empirical studies and considering theories of addictive behaviors, several theoretical models of the development and maintenance of IGD have been proposed. Recently, IGD has been included in the third section of the latest (fifth) edition of the Diagnostic and Statistical Manual for Mental Disorders (DSM-5) as a condition that requires additional research. Meanwhile, the World Health Organization (WHO) has included gaming disorder (both predominantly online and predominantly offline) in the ICD 11th revision. This progress dramatically raises academic discussions and public concerns for the importance of studying IGD. The precise neural mechanisms underlying the development, maintenance, and remission of IGD still require further investigations in order to better understand the phenomenon of IGD and to improve treatment outcome.

In this Research Topic, we begin with a review paper by Kimberly Young, a pioneer in the field of Internet addiction research, and Matthias Brand. Young and Brand summarize general aspects of IGD including diagnostic criteria and classification, and they also emphasize the Interaction of Person-Affect-Cognition-Execution (I-PACE) model, a comprehensive model which is based on empirical studies and which aims at inspiring future theory-driven research and new treatment protocols for IGD. Under the framework of Research Domain Criteria (RDoC), advocated by the National Institute of Mental Health, Kuss et al. review brain imaging studies of IGD. They report that gaming addicts have poorer response-inhibition, working memory, decision-making and emotion regulation, which is associated with reduced prefrontal cortex functioning, and conclude that deficiencies in the neural reward system is one key element of IGD, similar to the results found in individuals with substance-related disorders. Based on the recent functional Magnetic Resonance Imaging (fMRI) studies, Weinstein also finds that individuals with IGD show alterations in executive function, decision-making, behavioral inhibition and emotion regulation, which are similar to those reported for SUD. Weinstein also stresses that future studies need to investigate white matter density and functional connectivity in IGD to validate recent findings and to disentangle potential similarities and differences in neuro-chemical and neuro-cognitive brain circuits in IGD and co-morbid conditions such as ADHD and depression. Wei et al. conclude that the interaction of three systems, the impulsive system, the reflective system, and the interoceptive-awareness system plays a major role in IGD and argue that the development and maintenance of IGD is associated with a hyperactive “impulsive” system, a hypoactive “reflective” system, and is exacerbated by the interoceptive-awareness system.

Reward processing plays a critical role in adaptive behavior and has been consistently found impaired in SUD. Kim et al. report that individuals with an overuse of Internet games are more likely to fail to choose the response previously reinforced by symbolic (but not monetary) reward, which is accompanied by reduced neural responses to reward in the inferior parietal region and medial orbitofrontal/ventromedial prefrontal cortex. Wang et al. try to control potential effects of cue-familiarity and find that compared to recreational Internet game users, individuals with IGD show enhanced brain activity in the left orbitofrontal cortex and decreased activity in the right anterior cingulate cortex during processing of gaming-related cues. This seems to be linked to the high desire for game playing and the impaired ability in inhibiting the craving for gaming in subjects with IGD. Using ERPs techniques, Peng et al. present data showing that in individuals with IGD amplitudes in ERP component N170 (an index of early face processing) is decreased in response to neutral face expressions compared to happy face expressions, but no group difference during the processing of sad expressions and neutral expressions. They conclude that individuals with IGD may expect more positive emotions in the happy–neutral expressions context. Wang et al. explore impaired decision making using an intertemporal decision-making task among IGD. Compared to control subjects, the IGD group tends to pursuit immediate satisfaction, which is accompanied by reduced brain activations in the dorsolateral prefrontal cortex and bilateral inferior frontal gyrus. These findings indicate a deficiency in the ability of evaluating delayed reward and immediate satisfaction, and an impaired ability in impulse inhibition.

Three studies explore the neural evidence to show negative effects of longtime exposure to violent video game. Pan et al. use the amplitude of low-frequency fluctuations (ALFF) and fractional ALFF (fALFF) to quantify the group difference of spontaneous brain activity between a violent video game group and the control group but didn't find any group difference. Gao et al. further explore whether the exposure to violent video games (VVG) could change players' empathic responses to painful situations, and the results show that the perception of others' pain aren't significantly different in brain regions between violent video game group and non-violent video game group. The study by Szycik et al. did not find group differences in brain responses to emotional cues between excessive users of violent games and control subjects. Given that there are many theoretical and empirical evidence for the negative effect of longtime exposure to violent video game on children and adolescents' development, these results still warrant further investigations and this study inspires future research.

One study in this Research Topic also examines the association between alteration in brain structure and tendency to IGD. Using the index of gray matter volume (GMV), Pan et al. report that GMVs of the bilateral post-central gyri, the left precentral gyri, the left posterior midcingulate cortex, and the right middle frontal gyrus are negatively related to the tendency of IGD symptoms even after controlling for age, years of education and the time spending on online games. These results implicate that the GMVs of brain regions involved in sensorimotor processes and cognitive control are associated to IGD symptoms. Finally, the study by Müller et al. explores the associations between prenatal testosterone and both unspecified Internet addiction disorder and IGD. Digit ratio (2D:4D, digit ratio of the index to the ring finger) marker of the hand was used as a marker of prenatal testosterone. This study reports an association between lower digital ratio (2D:4D, right side, digit ratio of the index to the ring finger, i.e., >1 means lower prenatal testosterone) and higher symptoms of IGD. Moreover, this effect was particularly shown in female participants. These results implied that the 2D:4D marker might be an interesting biomarker for Internet addiction and warrants further studies.

In conclusion, the articles included in this Research Topic focused on several aspects of neurobiological mechanisms underlying IGD. The articles demonstrate that there is already empirical evidence for considering IGD a disorder due to addictive behaviors. However, more systematic and theory-driven studies including different subgroups and in particular longitudinal studies on the brain-behavior association in individuals with symptoms of IGD and other types of Internet-use disorders, are needed to better understand mechanisms of the development and maintenance of this addictive behavior and to optimize intervention techniques.

## Author contributions

J-TZ wrote the first draft of the manuscript. J-TZ and MB provided critical revision of the manuscript and important intellectual contributions. Both authors read and approved the submitted version.

### Conflict of interest statement

The authors declare that the research was conducted in the absence of any commercial or financial relationships that could be construed as a potential conflict of interest.

